# Loading capacity of dynamic knee spacers: a comparison between hand-moulded and COPAL spacers

**DOI:** 10.1186/s12891-019-2982-5

**Published:** 2019-12-21

**Authors:** Sook-Yee Chong, Lu Shen, Sandra Frantz

**Affiliations:** 1 Department of Orthopaedic Surgery, University Hospital Tuebingen, Tuebingen, Germany; 2 Faculty of Health Sciences, Oslo Metropolitan University, Oslo, Norway; 3 Institute of Applied Mechanics, University of Stuttgart, Stuttgart, Germany

**Keywords:** Dynamic knee spacers, Hand-mould spacers, Load, Axial displacement, Stiffness

## Abstract

**Background:**

The two-stage revision protocol represents the current gold standard for treating infected total knee replacement implants. Allowing early mobility with weight-bearing between staged procedures will enable early restoration to knee function. So, the mechanical performance of knee spacers is a key issue. Commercially available moulds are often used as they are easy to prepare and produce smoother surfaces of the articulating parts. However, they are costly, and only for single use. A cost-effective alternative is the surgeon-made hand-moulded spacers. In this study, we wanted to determine how the hand-moulded spacers will compare biomechanically with the commercially available COPAL spacers.

**Methods:**

Seven cadaveric knees were implanted with knee spacers fabricated using COPAL knee moulds. The same surgeon implanted eight cadaveric knees with hand-moulded spacers. In the first test protocol, an axial load was applied at 200 mm/min till failure. In the second test protocol, the knees were cyclically loaded in five steps of 1000 cycles each from 30-400 N, 30-600 N, 30-800 N, 30-1000 N, 30-1200 N at 1.5 Hz.

**Results:**

COPAL knee spacers demonstrated a maximum load and mean stiffness of 5202 (± 486.9) N and 1098 (± 201.5) N/mm respectively. The hand-moulded knee spacers demonstrated a mean stiffness of 4509 (± 1092.6) N and 1008.7 (± 275.4) N/mm respectively. The maximum axial displacement was 1.19 ± 0.57 mm and 0.89 ± 0.30 mm for specimens implanted with COPAL knee spacers and hand-moulded spacers respectively. The differences between COPAL and hand-moulded knee spacers were not statistically different.

**Conclusions:**

Our study demonstrated that dynamic knee spacers may be able to withstand more than the touch-down load permitted in previous studies, and this may allow more weight-bearing during ambulation. Previous studies have demonstrated that hand-moulded knee spacers have similar advantages to commercially available dynamic spacers with respect to mobility, pain, bone loss, and reinfection rate. Given that ambulation with weight-bearing up to 1200 N is permitted during rehabilitation, it may be more cost-effective to fabricate hand-moulded spacers in revision total knee arthroplasty.

## Background

The two-stage revision protocol represents the current gold standard for treating infected total knee replacement implants [1–5]. When an infected prosthesis is first removed, patients will receive an antibiotic-loaded cement spacer. The ideal duration for antibiotic therapy is not defined, but previous studies recommend a six- to twelve-weeks course [6–11]. When the infection is eradicated, a second surgery is performed to remove the spacer and re-implant a revision prosthesis. Muscle loss and soft tissue retraction during the time between procedures are common; they also prolong the second surgical stage and the recovery thereafter.

Dynamic spacers allow some knee movement between procedures, which may facilitate re-implantation and improves the range of motion after second-stage revision surgery [6, 12]. While the advantages of dynamic spacers in the treatment of infected total knee arthroplasty (TKA) have been widely described, there is no agreement about the best type of spacer to be used [13–20]. Dynamic spacers can be fabricated using different moulds, or may be purchased pre-packaged by the manufacturer. It appears the type of spacer implanted does not influence the infection eradication rate in TKA revision for infection; Among the various types of dynamic spacers in use (PROSTALAC, Hofmann technique, cemented moulds, and Spacer-K), control of infection does not appear to differ significantly between manufactured spacers and surgeon-made (or hand-moulded) dynamic spacers [21].

However, to prevent muscle atrophy, allowing early ambulation with weight-bearing between staged procedures will enable early restoration to knee function. With the use of the PROSTALAC antibiotic-loaded spacer (Depuy, Warsaw, IN), patients were allowed weight bearing and motion as tolerated by the joint before re-implantation of a revision prosthesis between 18 weeks and two years. After week eight postoperatively, 95% of the patients were allowed full weight bearing, without the use of assistive devices [3, 17, 22]. It should be noted that while PROSTALAC is made primarily of antibiotic-loaded bone cement, the articular surfaces are fabricated with stainless-steel on ultrahigh- molecular-weight polyethylene (PE) [7]. In other studies, full weight bearing was discouraged [23] when articulating components are fabricated only from cement, and cement-covered PE-fabricated components.

Commercially available moulds are often used during fabrication of dynamic spacers as they produce significantly smoother surfaces of the articulating parts and are easy to prepare [23–25]. However, the system is considered rather expensive since the moulds are only for single use [7, 26]. A commonly-used and cost-effective alternative is the hand-moulded spacers. They can be fabricated in any operating theatre without specific tools and moulds, and retain most of the advantages of dynamic spacers [27–29]. Another added advantage of hand-moulded spacers includes the possibility of adding higher doses of antibiotic in the bone cement [25, 30]. The main disadvantages of hand-moulded spacers are that they may not have reproducible mechanical characteristics [31], and the spacers are potentially unstable [14] so an extension brace or orthosis have to be worn during ambulation [2, 27, 32].

Since at least six weeks must pass between the two stages and the patient should be encouraged to weight-bear on the knee to prevent soft tissue contractures, the mechanical performances of dynamic knee spacers fabricated from bone cement is a key issue. Evaluation of the spacers’ ability to withstand loading during the six weeks’ rehabilitation period between the two-stage revision, is not yet present in literature. So the main aim in this study, is to biomechanically assess the loading capacity of implanted dynamic knee spacers fabricated from COPAL knee moulds (Heraeus Medical GmbH, Wehrheim, Germany), and compare them with hand-moulded spacers.

## Methods

Cadaveric specimens were purchased from Science Care, Phoenix, Arizona, USA. Ethics for the research protocol, procurement and use of cadaveric specimens were obtained from the ethics committee of the medical faculty of Uniklinik Tuebingen (proj nr: 006/2018B02) in accordance with the Declaration of Helsinki. COPAL bone cement (40 g per packet) impregnated with 1.0 g of gentamicin and 1.0 g of clindamycin (Heraeus Medical GmbH, Wehrheim, Germany) were used to fabricate both types of knee spacers. Skin and other soft tissues were first removed. One surgeon prepared all the specimens for implanting of knee prostheses [33]. Thereafter, the prosthesis was removed, taking precaution to ensure minimal bone loss. The dynamic cement spacers were then implanted securely with cement.

### COPAL knee spacers

**Fig. 1:**
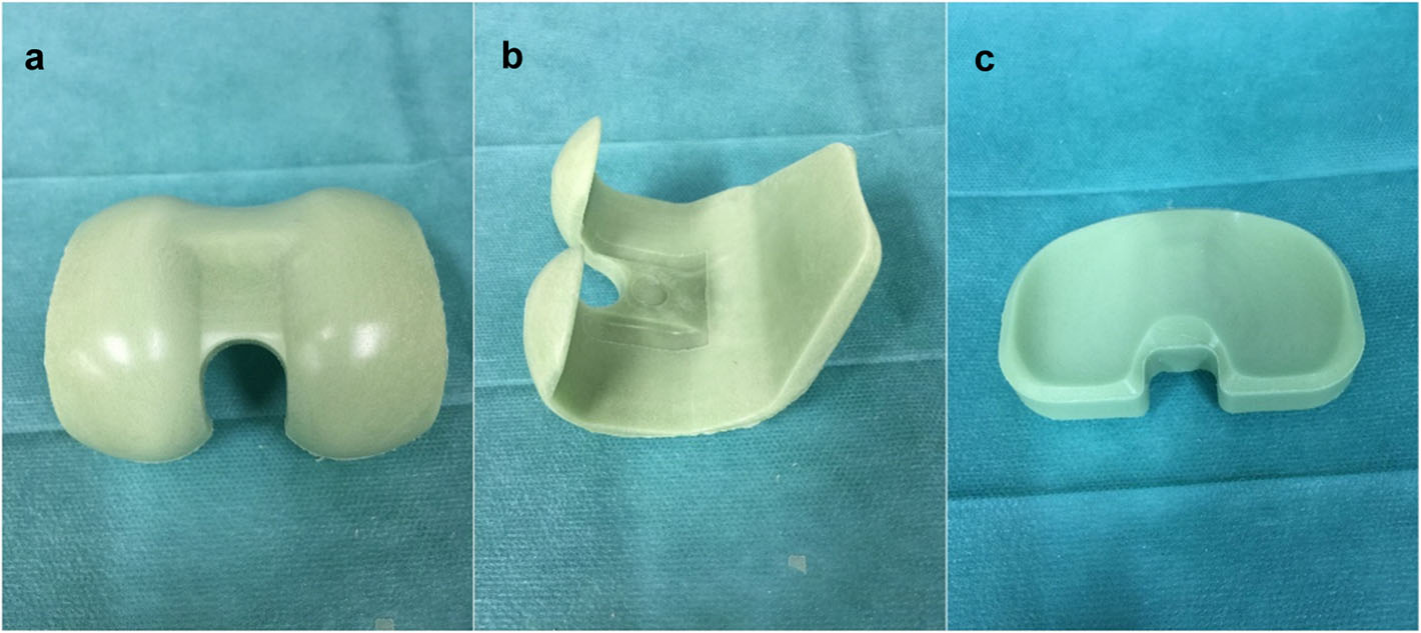
The femoral (**a**, **b**) and tibial components (**c**) of the knee spacer fabricated from COPAL knee moulds

**Fig. 2:**
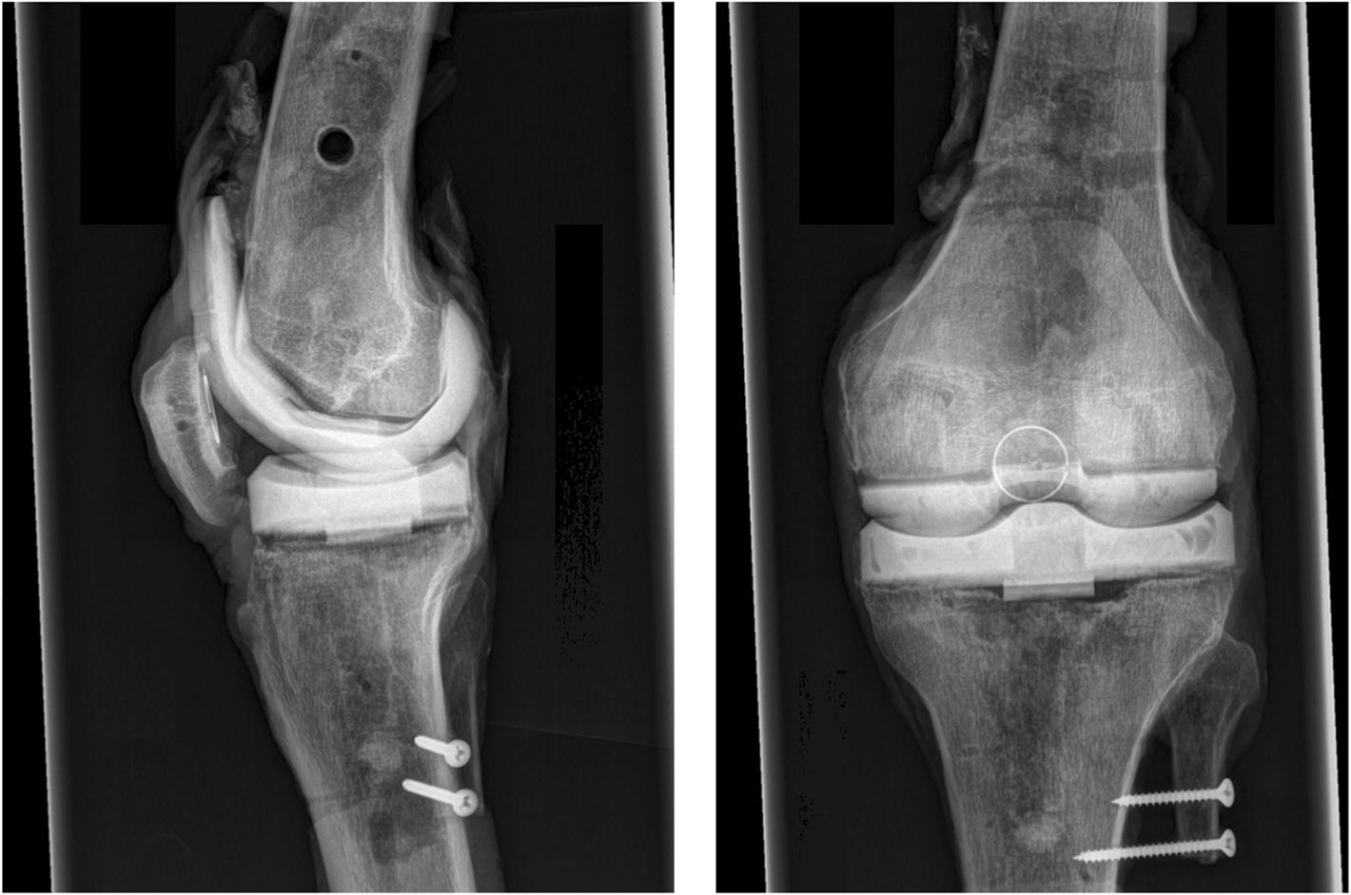
X-ray of one specimen with COPAL dynamic spacer before testing

Seven knees (out of eight) were implanted with COPAL knee spacers fabricated using COPAL knee moulds. The tibial plateau of one specimen was damaged during removal of the prosthesis, so it had to be removed from the study. Mould sizes and height of tibial component were determined by the same surgeon (Figs. 1, 2). One spacer was fabricated for each specimen. The spacer was implanted on the day of testing.

### Hand-moulded knee spacers

**Fig. 3:**
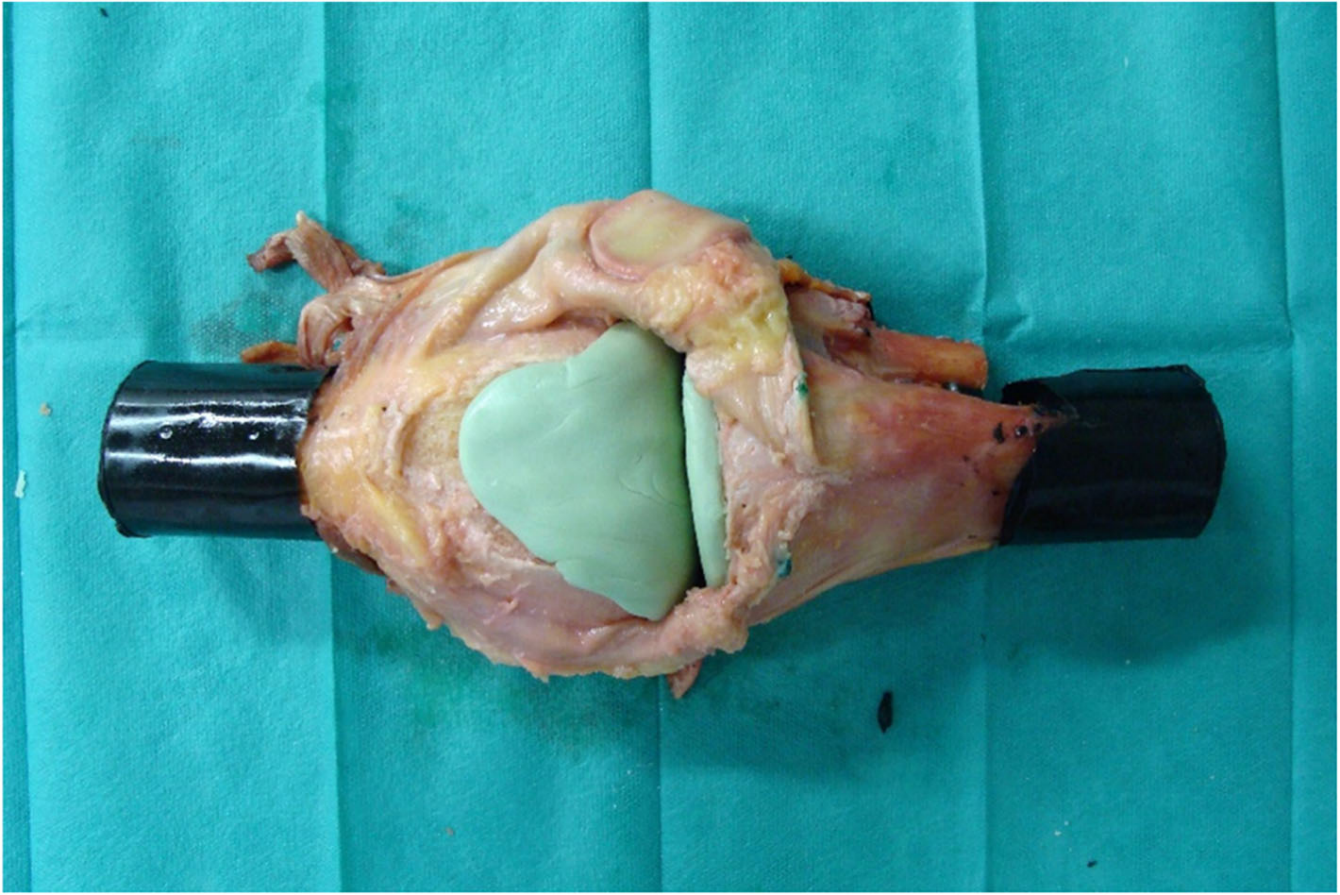
One knee specimen implanted with hand-moulded spacers

**Fig. 4:**
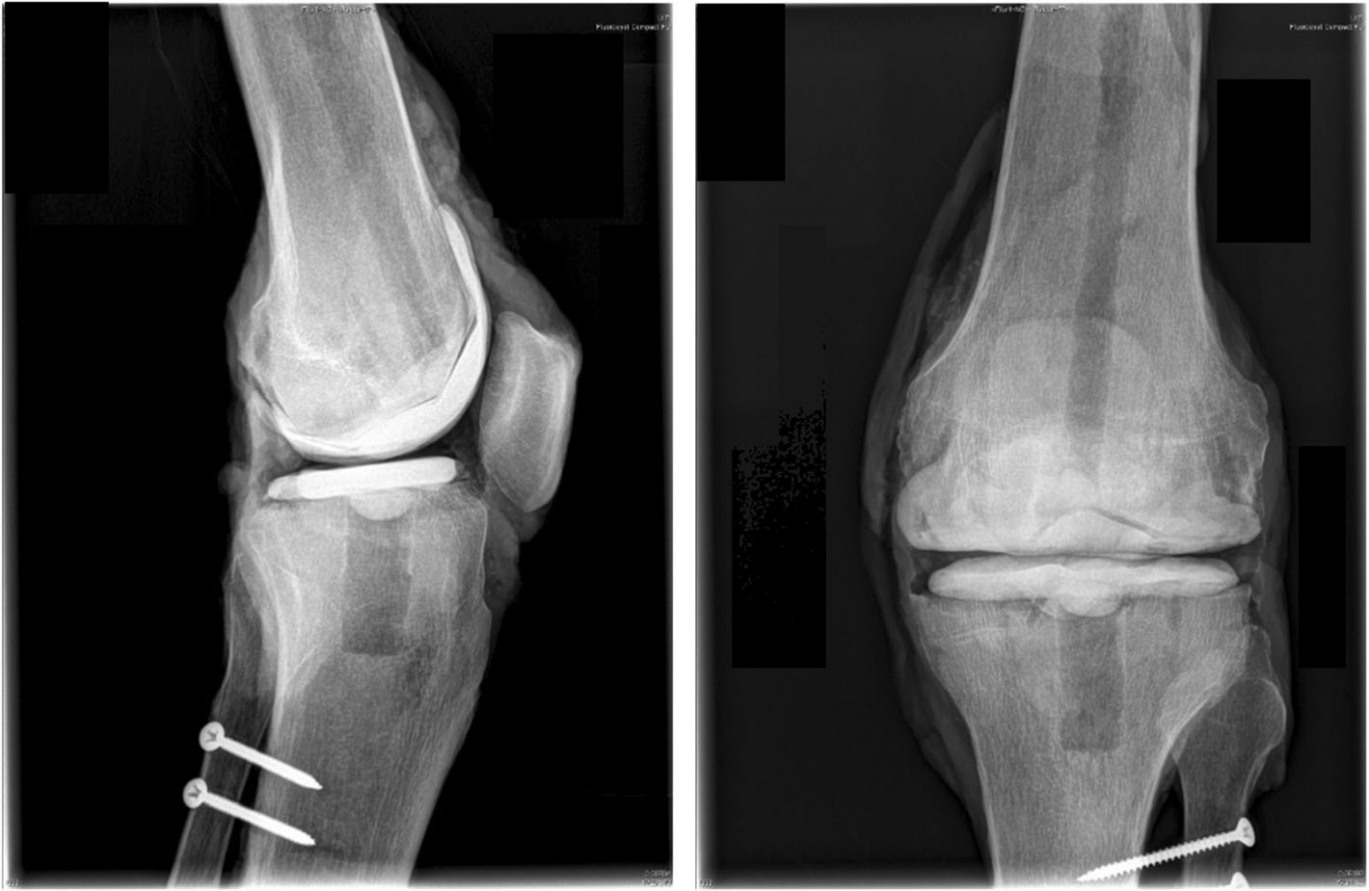
X-ray of one specimen with hand-moulded dynamic spacer before testing

Eight knees were implanted with hand-moulded spacers (Figs. 3, 4). All hand-moulded spacers were fabricated and implanted by the same surgeon. The spacer was fabricated directly onto the specimen and ensured that it adhered strongly to the bone surface. Fabrication was performed on the day of testing. One spacer was fabricated for each specimen. To ensure that the bone cement is completely polymerized, we waited two hours before performing the experiments.

### Experiments

**Fig. 5:**
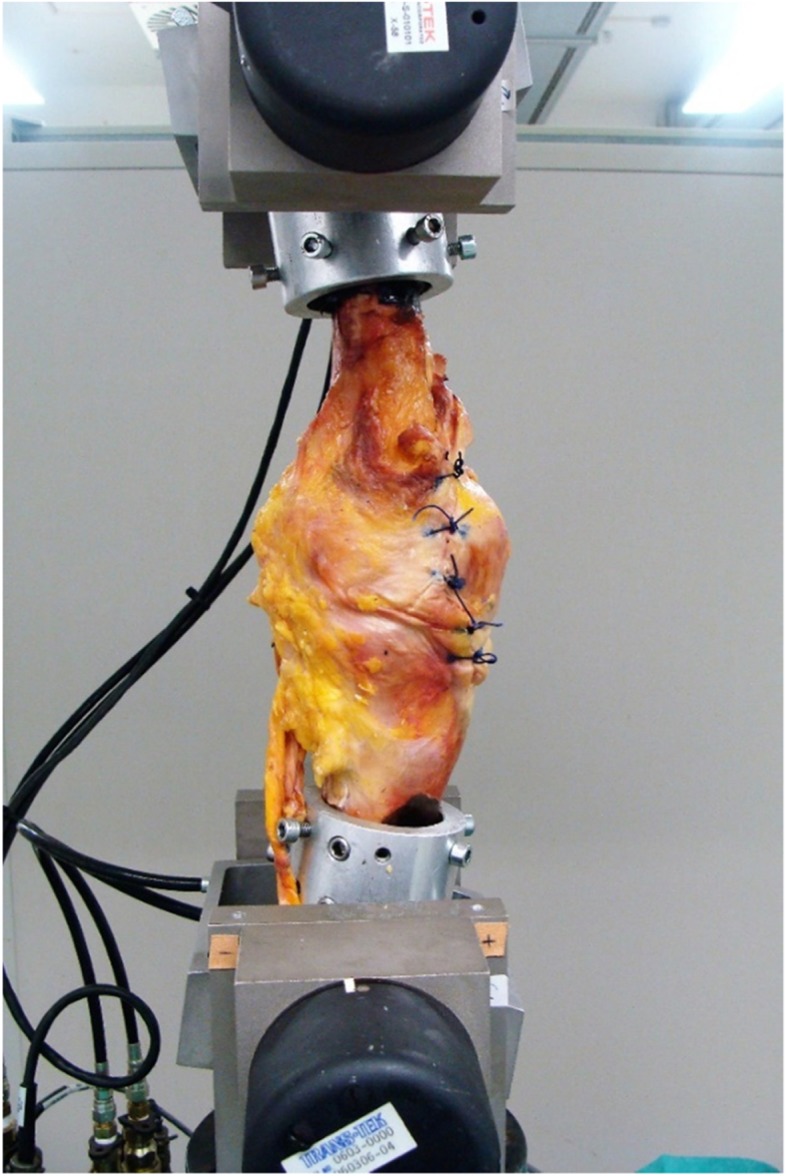
One knee specimen mounted at 10 degrees flexion between the upper and lower grips of the MTS machine

Experiments were performed on the MTS (MTS Systems, MN, USA) testing machine. All tests were performed at room temperature. Radiographic images of each specimen was performed prior testing, to ensure the integrity of the specimens. The femur and tibia ends were embedded and fixed into aluminum cylinders using bone cement. In the femoral clamp, the femur was mounted to a jig which is tilted by six degrees angle with respect to the normal, resembling the physiological valgus [34]. Each knee was placed in ten degrees flexion which was adopted from Wan et al., 2012 [25] since postoperative patients in the Uniklinik Tuebingen were to keep their knees immobilised in extension for six weeks (Fig. 5).

**Fig. 6:**
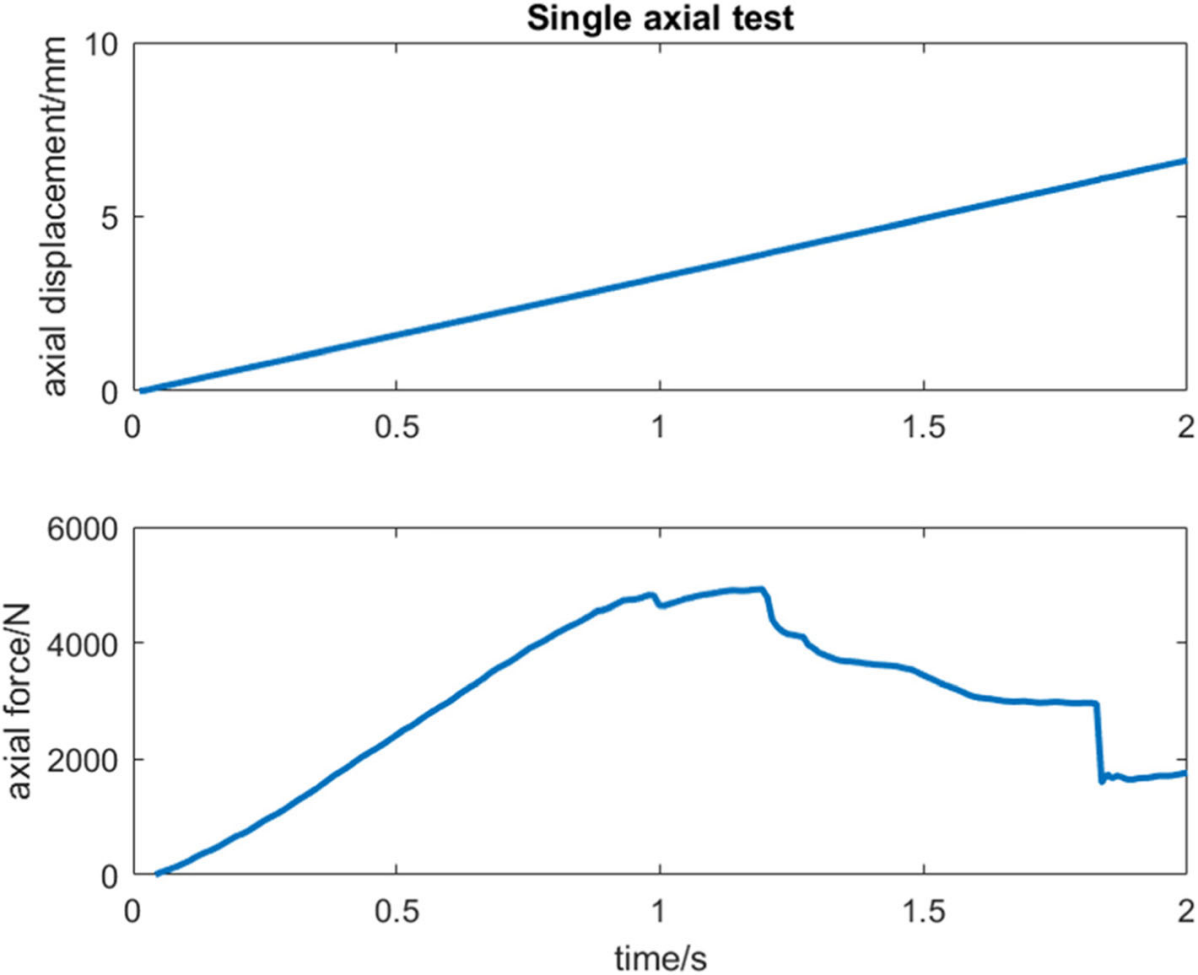
First protocol where the knee specimen underwent a single axial loading test

**Fig. 7:**
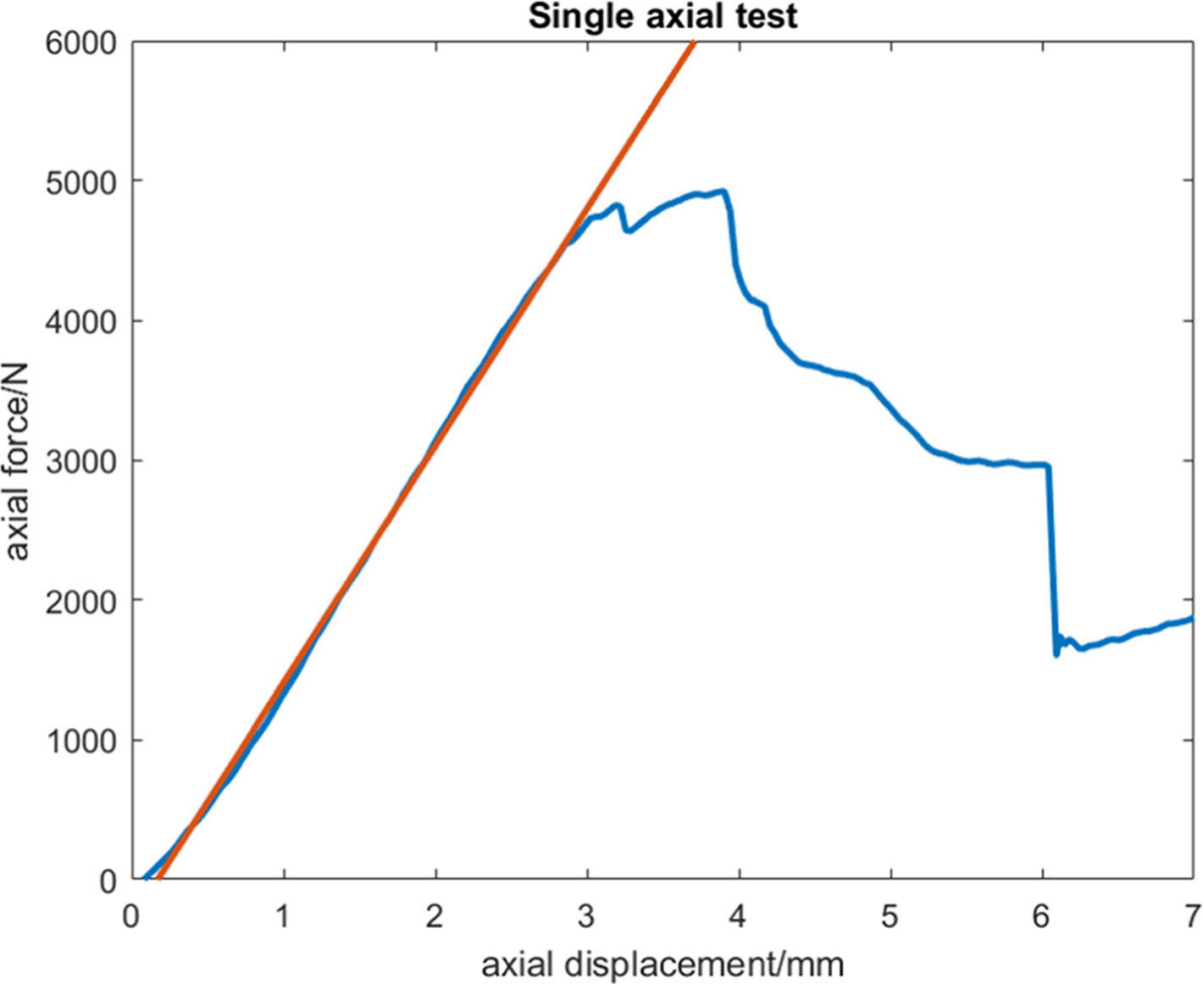
Load-displacement curve with stiffness calculated from linear gradient (red line)

The specimens were subjected to two different loading protocols. In the first protocol, the specimens underwent a single axial loading test. This was to access the mechanical reliability of the spacer in a situation when the patient does not use crutches or any support during rehabilitation. During testing, an axial load was applied with a loading rate of 200 mm/min on the knee specimen [35] (Fig. 6). After testing, maximum load and stiffness were determined. Maximum load was defined to be the highest measurable load during the single axial loading test. Stiffness was defined as the gradient of the linear region of the load-displacement curve, determined from least-squares fitting (Fig. 7).

**Fig. 8:**
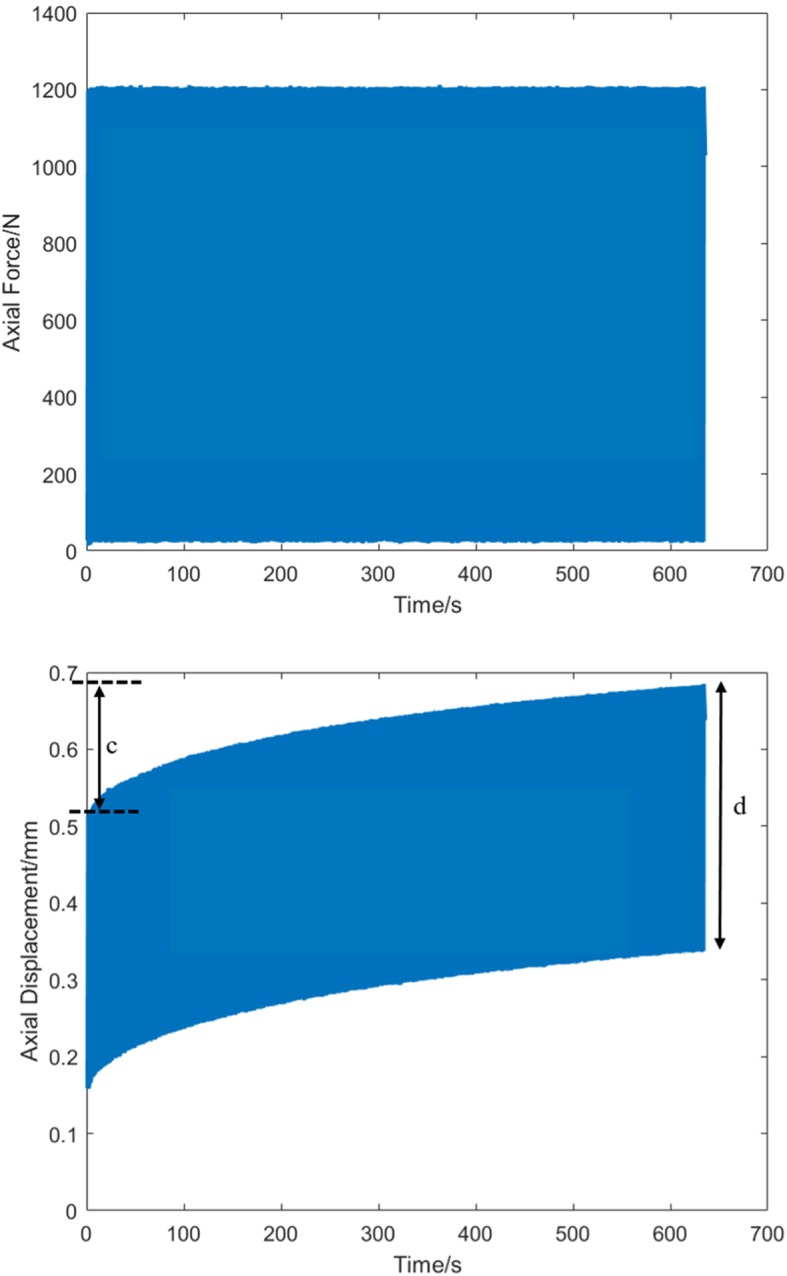
Axial force (top) and displacement (bottom) of 30-1200 N cyclic loading test of one specimen implanted with COPAL spacer. Axial displacement (d) at the 1000^th^ cycle was determined. c is the maximum amount of deviation from the first cycle

In the second protocol, the specimens underwent a step-wise cyclic loading test, adapted from Weimann et al., 2013 [35]. A preload of 30 N was first applied. Then, the specimens were cyclically loaded in five steps: 30–400 N, 30–600 N, 30–800 N, 30–1000 N, and 30–1200 N (Fig. 8). This protocol represents a reasonable amount of knee loading experienced during rehabilitation [35]. There were 1000 cycles in each step. Cyclic loading was performed at a loading frequency of 1.5 Hz; This was the average frequency calculated from the test protocol of 1.3 Hz suggested by Weimann et al., 2013 [35] and the average walking frequency of 1.8 Hz reported by Pachi and Ji 2005 [36]. Axial displacement (*d*) after every 1000th cycle, and changes to the axial displacement (*d*) at each cycle was also determined. Since contact mechanics at different regions of the knee are affected by loading, *d* would describe the relative translation of the tibia with respect to the femur during applied axial load. After testing, failure was visually monitored by the surgeon and in radiographic images. Results were tested for statistically significant differences (two-tailed, *p* < 0.05) using an unpaired t-test. Levene’s test was used to assess the equality of variances between groups.

## Results

From three specimens implanted with COPAL knee spacers, the result of the axial load-to-failure test showed a maximum load and mean stiffness of 5202 (± 486.9) N and 1098 (± 201.5) N/mm (Table 1). Four specimens implanted with hand-moulded knee spacers, exhibited a maximum load and mean stiffness of 4509 (± 1092.6) N and 1008.7 (± 275.4) N/mm respectively (Table 1). Mean difference for maximum load was 692.2 (± 687.8) N [95% confidence interval (95% CI): − 1075.9, 2460.4]. Mean difference for stiffness was 89.6 (± 189.8) N/mm [95% CI: − 398.2, 577.5].

**Table 1:** Results of specimens that underwent the axial load-to-failure test, and equality of variance

	Mean ± SD			variance
	COPAL (*n* = 3)	hand-moulded (n = 4)	*p*-value	p-value
Maximum Load (N)	5202.0 ± 486.9	4509.7 ± 1092.6	0.32	0.36
Stiffness (N/mm)	1098.3 ± 201.5	1008.7 ± 275.4	0.64	0.56

There was a time-dependent axial displacement for all specimens during cyclic loading (Fig. 8). However, changes of axial displacement (*d*) at every cycle was not significantly different (refer to Additional file 1: Figure. S1). So, axial displacement after every 1000th cycle was represented in Table 2 for both types of knee spacers. The maximum mean displacement of four specimens implanted with COPAL knee spacers after 5000 cycles was 1.19 ± 0.57 mm (Table 2). The mean axial displacement was 0.36 (± 0.14) mm, 0.63 (± 0.30) mm, 0.83 (± 0.40) mm, 1.01 (± 0.44) mm, and 1.19 (± 0.57) mm, with cyclic loading forces ranging from 30 N to 400 N, 30 N to 600 N, 30 N to 800 N, 30 N to 1000 N, and 30 N to 1200 N respectively. There was a mean of 0.19 (± 0.04) mm increase with every additional load of 200 N.

**Table 2:** Results of mean displacement *d* (in mm) of specimens underwent cyclic loading test, and equality of variance

	Mean ± SD			variance
Cyclic load	COPAL (*n* = 4)	hand-moulded (*n* = 4)	*p*-value	*p*-value
[30 400]	0.36 ± 0.14	0.36 ± 0.20	0.99	0.56
[30 600]	0.63 ± 0.30	0.48 ± 0.19	0.42	0.33
[30 800]	0.83 ± 0.40	0.65 ± 0.30	0.48	0.67
[30 1000]	1.01 ± 0.44	0.84 ± 0.43	0.62	0.96
[30 1200]	1.19 ± 0.57	0.89 ± 0.30	0.36	0.26

The maximum mean displacement of four specimens implanted with hand-moulded knee spacers after 5000 cycles was found to be 0.89 ± 0.30 mm (Table 2). The mean axial displacement was 0.36 (± 0.20) mm, 0.48 (± 0.19) mm, 0.65 (± 0.30) mm, 0.84 (± 0.43) mm, and 0.89 (± 0.30) mm, with cyclic loading forces ranging from 30 N to 400 N, 30 N to 600 N, 30 N to 800 N, 30 N to 1000 N, and 30 N to 1200 N respectively. There was a mean of 0.13 (± 0.08) mm increase with every additional load of 200 N.

## Discussion

During the tests, the tibia was fixed in the MTS machine, which did not represent physiological conditions during knee loading. While the testing protocol of the single axial and cyclic loading in this study may not reflect the complexity of in-vivo loading occurring at the knee joint, applications of these simple loading patterns can provide an insight into possible spacer failure during repetitive loading. In addition, only compression tests were performed in this study. Mechanical characteristics may be different in other loading modes. Transverse loading or side load capacity was not considered in this study, though it is usually smaller than the normal axial load capacity [37].

It should be noted that the highest measurable load and stiffness were determined in-vitro, without any active muscle forces and passive soft tissues. A higher joint compressive load will be expected when muscle forces act to control joint motion during translation and rotation. The load sharing properties between muscles and ligaments will also provide the knee its stability at a higher load. In this study, no significant differences were found in the highest measurable load (Table 1) though the spacers were dislodged after the single axial loading test. Loosening of the spacers would be considered a failure since this would require another surgery. The exact load at which the spacers were dislodged is unknown in this study. No significant differences were also found in the stiffness during the single axial loading test (Table 1). While the COPAL spacers were fabricated from moulds, the hand-moulded spacers were custom-made and dependent on the surgeon, which may result in a wider standard deviation. Since both COPAL and hand-moulded spacers were fabricated from the same material, variability in stiffness may also be due to differences between the specimens and attachment at the spacer-bone interface. During the single axial loading test, the spacers were not broken but dislodged from the knee joints. In other words, the spacer may not have reached its fracture point, but there was a failure to transfer loading between the spacer and the bone when an axial load was applied. Nevertheless, we acknowledge that a report of no significance differences in our study could be a Type 2 error.

Novitskaya et al., 2014 [38] found that compressive creep loading of bone samples at the proximal tibia resulted in bending of the trabecular bone, leading to permanent deformation. We postulated that the time-dependent response in the axial displacement may be a result of creep behaviour of the trabecular bone. Since the axial displacement is small (*d* < 2 mm, *c* = 0.22 ± 0.21 mm) (Fig. 8), it has not affected implant alignment [38], and has not resulted in loosening of the spacer. However, prolonged testing at higher loads may lead to loosening of the spacer, previously found in hip implants [39, 40],

Axial displacement in this study (Table 2) was larger than the 0.6–1.2 mm reported in intact knees [41], but within 0.98–3.10 mm in meniscectomized knees during compression loading up to 1000 N [42–44]. The wider range reported in meniscectomized knees could be due to differences of the joints tested or in the experimental setups. In this study, there were no signs of failure for both COPAL and hand-moulded spacers as determined visually by the orthopaedic surgeon, and no structural damage was found in radiographic images. The knee spacers were not dislodged from the knee joints, nor were they broken. So both COPAL and hand-moulded knee spacers could withstand weight-loading up to 1200 N. Nevertheless, we acknowledge that the absence of any visible damage or signs of failure during the cyclic loading test, is not necessarily related to permanent damage at the knee joint. There may be other damage to bone and soft tissues during in-vivo loading.

Few studies on dynamic knee spacers have reported the allowable loading capacity of these spacers. MacAvoy et al., 2005 [45] permitted full weight-bearing with the use of crutches or walker during ambulation with a hinged knee brace. Other studies allowed touch-down or partial weight bearing, but no more information was provided [1, 4, 7, 20, 23, 24, 46–51]. Hofmann et al., 1995 [52] and Hofmann et al., 2005 [22] allowed 50% of body weight on the affected knee with the use of crutches or a walker. Wan et al., 2012 [25] encouraged the patients to gently load bear to 312 N. Kohl et al., 2011 [26] instructed the patients to partial weight bear to 20 kg (196 N) 48 h after surgery. Our study demonstrated that both types of dynamic spacers may be able to withstand more than the touch-down load previously allowed in other studies, and this may permit more weight-bearing during ambulation for patients during the six weeks of rehabilitation.

A patient’s ability to bend the knee increases his or her quality of life between stages [4], especially if a long period of antibiotic therapy is necessary to eradicate the infection. In this study, the knee specimens were kept at an angle of 10° [25] during cyclic loading after consultation with the surgeon; When a dynamic spacer is implanted, patients in the Orthopaedics department in Uniklinikum Tuebingen kept their knees in an immobilizer in full extension for six weeks prior to the second-stage operation. Previous studies suggested a continuous passive motion (CPM) limited to 90° of flexion for patients after the first stage knee replacement surgeries, and revision surgeries [23, 32, 46, 50–52], to enable continued mobilization of soft tissues and the prevention of contractures. In this study, CPM was not considered. Accordingly, in our study, we cannot compare the allowable range of motion nor the loading capacity at different knee angles of both COPAL knee spacers and the hand-moulded knee spacers. Nevertheless, a comparable range of motion up to 104°- 107° was reported in previous studies [27, 50, 52, 53].

To ensure consistency, only one surgeon fabricated all preformed and hand-moulded spacers in this study. However in the clinics, the preparation of hand-moulded spacers can be custom-made by different surgeons, resulting in varied mechanical characteristics [31]. Other factors such as the surgeon’s experience with the fabrication of spacers and the amount of bone loss and scar tissue, will contribute to the successful outcome in using a spacer. So, the utilisation of one surgeon in this study is a limitation in extending these results to clinical practice. This research is also limited by a small sample size. We are compelled to restrict the sampling size due to limited availability of cadaveric specimens. In addition, specimens, which were examined clinically by the surgeon and in radiographs, were excluded if any degeneration and deformities were observed.

One major failure mechanism is loosening of the spacer due to loss of fixation at the spacer-bone interface. Since one possible cause of this loosening is dependent on the applied load [38], cyclic loading was increased step-wise to 1200 N. This maximum load was chosen after considering that the patient will be walking with the aid of crutches during rehabilitation. However, another limitation of this study is not cyclically loading the knee spacers to failure, which follows previous advice that full weight bearing should be discouraged [10, 23]. While knee spacers can also be fabricated with metal or PE articulating surfaces to enable a higher loading capacity, it should be emphasized that the main aim of a spacer is the eradication of infection. Metal and PE surfaces have no antibiotic protection, and thus, have the potential for re-infection [2, 20, 31]. In a pilot test, the femoral component in both the COPAL knee spacer and hand-moulded spacer loosened or broke (refer to Additional file 1: Figure. S2) when cyclic loading was performed at 2600 N. Further study focusing on fatigue loading of the knee spacers is envisioned, considering that cement spacers may exhibit abrasive wear after a few weeks [16, 54, 55].

## Conclusion

It has been argued that while the COPAL knee spacer is expensive and discarded after six weeks, expenses from a prolonged hospitalisation due to immobilisation and complications are significantly more. Savings from an earlier discharge from hospital can outweigh the increased cost of the use of the moulding system [17]. So far, hand-moulded knee spacers have similar advantages to commercially available dynamic spacers with respect to mobility, pain, bone loss, and reinfection rate [28, 50, 53, 56]. In this study, we have also demonstrated that the loading capacity between COPAL and hand-moulded knee spacers were not statistically different. However, given the small sample size, we nevertheless acknowledge the possibility of a Type 2 error. Given that ambulation with weight-bearing up to 1200 N is permitted during rehabilitation, it may be more cost-effective to utilise hand-moulded spacers in revision total knee arthroplasty.

## Supplementary information

**Additional file 1: Figure S1.** Mean and standard deviation of changes in axial displacement (d) during cyclic loading [30 1200 N] of all specimens implanted with COPAL knee spacers. **Figure S2.** Broken femoral component of COPAL knee spacer of one specimen during cyclic loading to 2600 N.

## Data Availability

All relevant data are within the manuscript, tables and its supplementary files. Data of human subjects are available from the University Hospital Tübingen for researchers who meet the criteria for access to confidential data, as stated in the ethics approval by the University Hospital Tübingen (project nr: 006/2018B02).

## Abbreviations

CPM: Continuous passive motion
PE: Ultrahigh- molecular-weight polyethylene
SD: Standard deviation
TKA: Total knee arthroplasty
