# Supplementary material for: Loading capacity of dynamic knee spacers: a comparison between hand-moulded and COPAL spacers
Source: BMC Musculoskelet Disord. 2019 Dec 21;20:613. doi: 10.1186/s12891-019-2982-5 (PMC6925492; doi:10.1186/s12891-019-2982-5)
Supplement: Supplementary file 1 — Additional file 1: Figure S1. Mean and standard deviation of changes in axial displacement (d) during cyclic loading [30 1200 N] of all specimens implanted with COPAL knee spacers. Figure S2. Broken femoral component of COPAL knee spacer of one specimen during cyclic loading to 2600 N. [file 12891_2019_2982_MOESM1_ESM.pdf]

Supplementary material

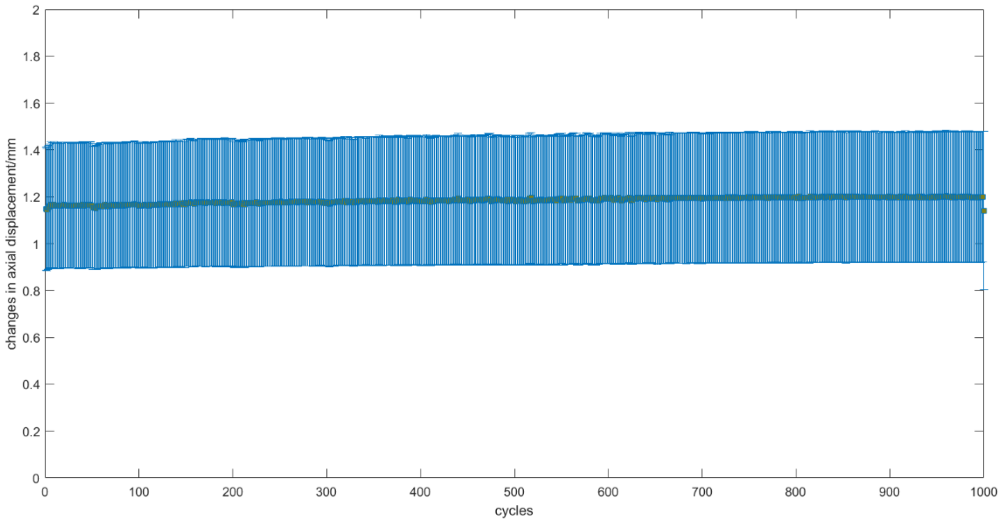

Figure S1: Mean and standard deviation of changes in axial displacement (*d*) during cyclic loading [30 1200 N] of all specimens implanted with COPAL knee spacers.

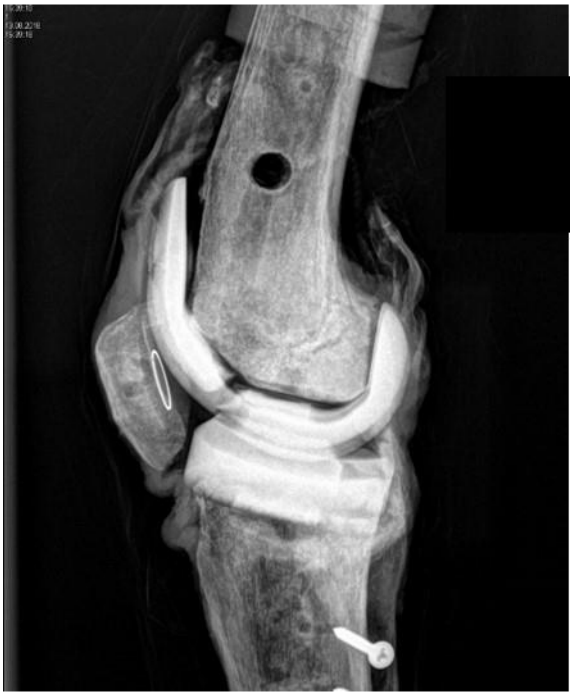

Figure S2: Broken femoral component of COPAL knee spacer of one specimen during cyclic loading to 2600 N
